# GENERALIZED LICHEN NITIDUS IN CHILDHOOD

**DOI:** 10.4103/0019-5154.44794

**Published:** 2008

**Authors:** Vandana Mehta, C Balachandran

**Affiliations:** *From the Department of Skin and STD, Kasturba Medical College, Manipal, Karnataka, India*

Sir,

Lichen nitidus was first described by Pinkus in 1901. It is an uncommon, chronic, papulosquamous eruption characterized by multiple, 1–2 mm, flesh-colored, shiny, dome-shaped papules. Although asymptomatic, it may sometimes be associated with pruritus.^1^ It classically involves the genitalia, upper extremities, chest and abdomen; in rare instances, it may become generalized.^2^ There appears to be no obvious racial or sex predilection; however, majority of cases are found in children and young adults. We present a young girl with a 4-year history of unremitting generalized lichen nitidus.

A 10-year-old girl presented with a 4-year history of an asymptomatic eruption that first appeared over the hands and upper extremities and gradually spread to involve the lower limbs and trunk. The patient was otherwise healthy, and there was no intake of any prior medications or a similar family history. Clinical examination revealed numerous 1–2 mm shiny and skin-colored papules over the hands, forearms, feet, legs and abdomen, particularly around the umbilicus with multiple foci of koebnerization apparent on the forearms (Figs. 1–4). The face, oral mucosa, nails, palms, soles and anogenital area were not involved. Biopsy findings from one of the papules showed a focal granulomatous lymphohistiocytic infiltrate in the papillary dermis immediately subjacent to the epidermis clutched by the elongated rete pegs, thereby producing a “claw clutching a ball” picture. This confirmed the clinical diagnosis of lichen nitidus (Fig. 5). The patient was initially started on potent topical cortiscosteroids and was provided with the option of NB-UVB twice weekly; subsequently, she was lost for follow-up.

**Fig. 1:**
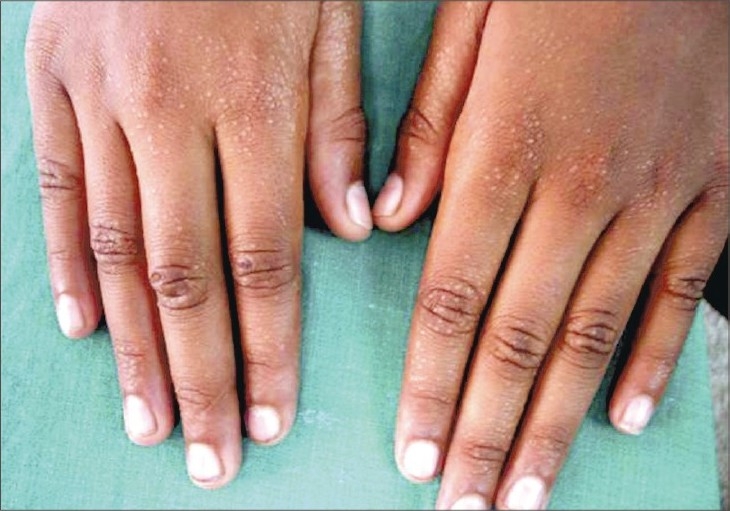
Skin-colored shiny papules on hands

**Fig. 2:**
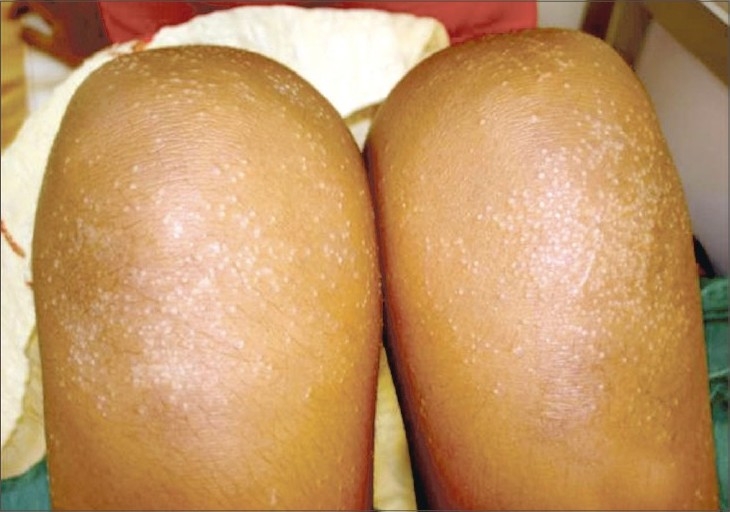
Shiny papules on knees

**Fig. 3:**
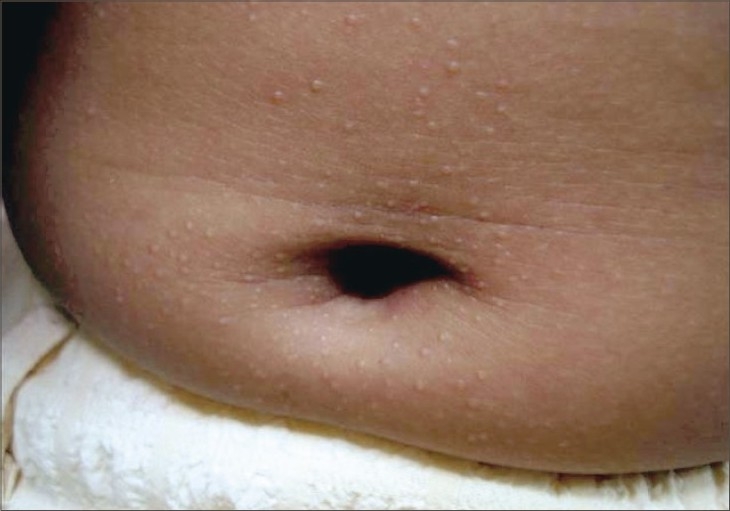
Shiny papules on abdomen

**Fig. 4:**
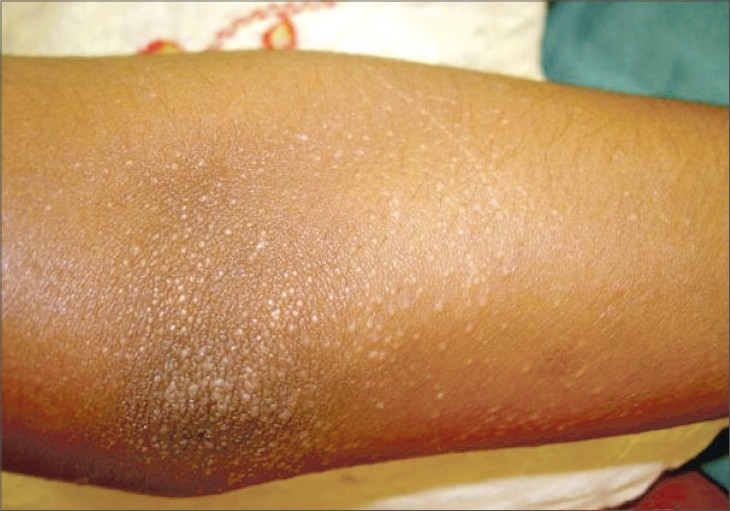
Foci of koebnerization on forearm

Generalized lichen nitidus is quite rare in children.^3^ In addition, several other uncommon variants that have been described include confluent, vesicular, hemorrhagic, familial, palmar and plantar, follicular, perforating and linear forms. Clinical instances of oral and nail involvement have also been reported in literature.^4^

The controversy regarding the etiology of lichen nitidus and its possible relationship to lichen planus is still unresolved. Some authors suggest that lichen nitidus represents a variant of lichen planus.^5^

Since lichen nitidus is usually asymptomatic and resolves without any sequelae, no treatment is required in most cases. However, the clinical course of generalized lichen nitidus is unpredictable with most patients experiencing spontaneous resolution several years after the onset of disease. Systemic and topical corticosteroids, dinitrochlorobenzene, diphenylcyclopropenone immunotherapy, astemizole, itraconazole, isoniazid, psoralen and ultraviolet A (PUVA) and narrow band ultraviolet B (NB-UVB) phototherapy have all been tried in its treatment, with PUVA, NB-UVB and astemizole being especially useful for generalized forms.^6^

## References

[1] Chen W, Schranm M, Zouboulis CC (1997). Generalised lichen nitidus. J Am Acad Dermatol.

[2] Maeda M (1994). A case of generalised lichen nitidus with Koebner's phenomenon. J Dermatol.

[3] Al-Mutairi N, Hassanein A, Nour-Eldin O, Arun J (2005). Generalised lichen nitidus. Pediatr Dermatol.

[4] Arizaga AT, Gaughan MD, Bang RH (2002). Generalised lichen nitidus. Clin Exp Dermatol.

[5] Stankler L (1967). The identity of lichen planus and lichen nitidus. Br J Dermatol.

[6] Do MO, Kim MJ, Kim SH, Myung KB, Choi YW (2007). Generalised lichen nitidus successfully treated with narrow band UVB phototherapy: two cases report. J Korean Med Sci.

